# Poly(N,N-dimethylaminoethyl methacrylate) as a bioactive polyelectrolyte—production and properties

**DOI:** 10.1098/rsos.230188

**Published:** 2023-09-20

**Authors:** Dawid Stawski

**Affiliations:** Institute of Materials Science of Textiles and Polymer Composites, Lodz University of Technology, Żeromskiego 116 str, 90-924 Lodz, Poland

**Keywords:** poly(N,N-dimethylaminoethyl methacrylate), bioactive polymer, copolymers

## Abstract

Poly(N,N-dimethylaminoethyl methacrylate) is a polyelectrolyte with many important chemical and physical properties and, above all, offers a wide range of interesting biological properties. Currently, research on this polymer is ongoing in several centres around the world. The process of polymerizing the monomer is not easy, as there are difficulties in obtaining a product with repeatable properties. This work collected and described most of the currently known and used polymerization methods of N,N-dimethylaminoethyl methacrylate, taking into account the type of method, the solvent used, the initiator, as well as the process temperature and the average molecular weight of the polymer obtained. The most important properties of the discussed polymer, such as solubility, bioactivity, hydrophilicity, cytotoxicity, conductivity, and thermal and hydrodynamic parameters, are discussed on the basis of the available scientific literature. This work aims, among other things, to increase the possibility of using poly(N,N-dimethylaminoethyl methacrylate) as a material in advanced practical applications. Therefore, various methods of applied use of the polymer in question have also been described so far. Copolymers of the N,N-dimethylaminoethyl methacrylate are now too large a collection to fit in a single publication. Therefore, only the most interesting examples were cited in this work.

## 1. Introduction

Electrolytes are chemical compounds that dissociate into ions dissolved in water, giving water the ability to conduct electrical current. Conduction of current through electrolyte solutions is based on the fact that ions wandering in an electric field carry electrical charges with them. This conduction is combined with the transport of matter, which causes changes in concentrations in solutions. Electrolytes are conductors of the second kind (metallic conductors are considered to be first-type conductors).

Polyelectrolytes, on the other hand, are ionic polymers in which each mer or most of them contain an ionized lateral group. Polyelectrolytes are divided into groups due to their origin, nature and power of the chemical groups. Taking into account the origin, synthetic, natural and natural modified polyelectrolytes are distinguished. From the point of view of the nature of the ion groups, we can divide polyelectrolytes into two groups:

— anionic polyacids and their salts, commonly containing carboxyl or sulfon groups;
— cationic polybases containing the most common amino groups;
— polyampholytes—polyelectrolytes composed of macromolecules containing both anion and cation groups or appropriate groups capable of ionization. In polyampholytes, ion groups with opposite loads, which are part of the side groups, are part of the same or different monomer. If ion groups are part of the same side group, then such polyelectolytes are called round-the-line polymers.

In anionic polyelectrolytes, the chain is, therefore, electrostatically neutralized by positive counterions, while in cationic polyelectrolytes, the chain is a polycation electrostatically neutralized by mobile anions.

Polyelectrolytes are obtained by various methods:

— by a polymerization of charged monomers;
— by modifying non-ionic polymers, analogous to the reactions to which low-molecular compounds are subjected to obtain acids or bases;
— by grafting ionic monomers on inert polymers.

The best homogeneity of the polyelectrolyte is obtained using the polymerization method. However, the polymerization of strongly acidic or strongly basic monomers can present some difficulties due to the ionic charge. In such a case, the reaction medium should be selected accordingly. In reactions carried out on inert polymers, the greatest difficulty is obtaining a homogeneous solution of the reactants and the polymer, especially because the reaction properties change the properties of the solution—it changes hydrophilic from hydrophobic [1].

Also, grafting reactions can lead to the formation of heterogeneous products. Many polyelectrolytes belong to the group of vinyl polymers, and the type of substituent determines their acid or base strength.

### 1.1 Anionic polyelectrolytes

Compounds containing a sulfone group are strongly acidic (pKa ≈ 1). The most typical for this group is poly(styrene sulfonate) (PSS). Its polymerization is easier when the monomer is a salt and the dielectric constant of the environment is low. Examples of strongly acid polyelectrolytes containing sulfonate groups are given below.

PSS


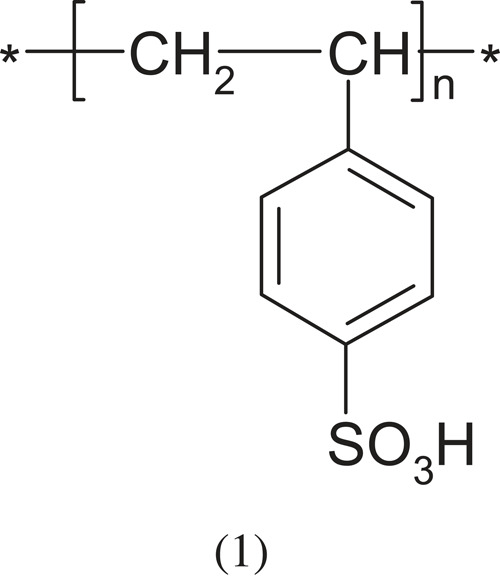


Poly(vinylsufonic acid)


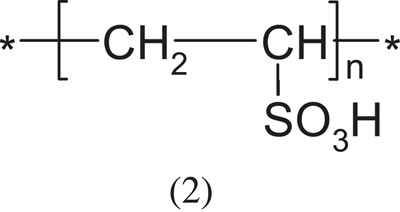


poly(sulfoethylmethacrylic acid)


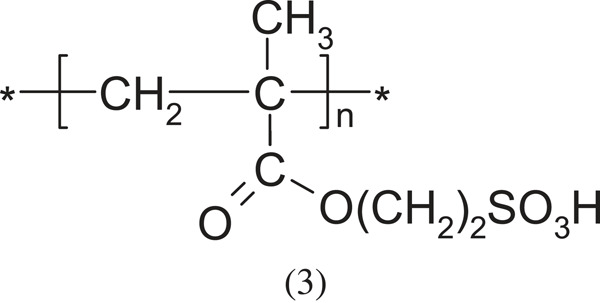


Polyelectrolytes with a carboxyl group are weakly acidic (pKa ≈ 6). The most representative of this group are:

poly(acrylic acid)


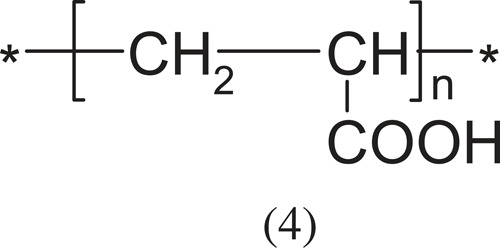


and poly(methacrylic acid).


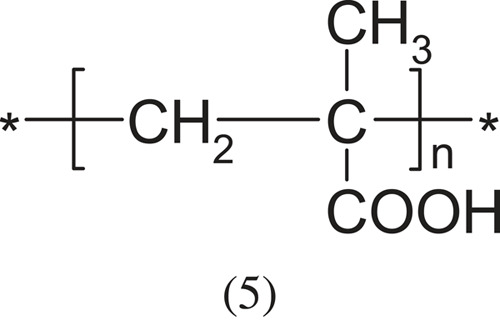


The above polyacids with a carboxyl group have played a huge role in the science of polyelectrolytes [1]. Acrylic acid and methacrylic acid polymerize spontaneously, and the polymerization is by a free radical mechanism. It can take place in an aqueous or non-aqueous environment or in suspension against peroxides or azo compounds.

Among other acidic polyelectrolytes, vinyl polymers with phosphoric acid groups should be mentioned:


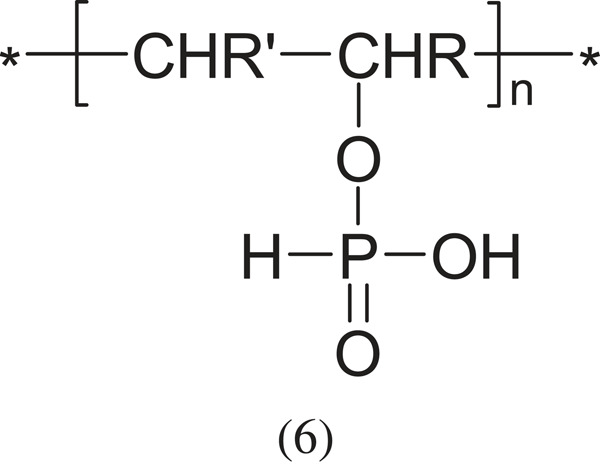


hypophosphorous acid


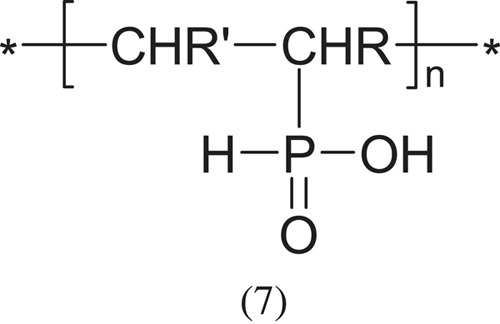


and phosphorous acid,


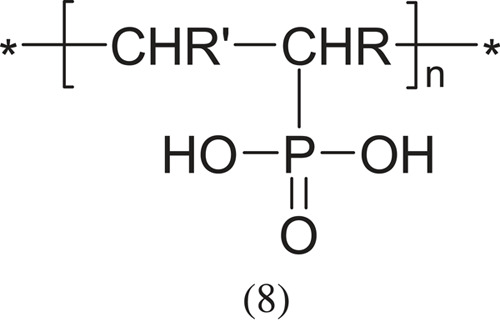


which are much less known and researched, mainly due to the smaller application possibilities (R and R'- appropriate group: -CH < , -CH2-, -CH3 or other).

### 1.2. Cationic polyelectrolytes

Cationic polymers are obtained from low molecular weight amines: primary, secondary, tertiary or quaternary ammonium salts. The methods of synthesizing them are more complex than that of anionic polymers. The highly basic character is given to these polyelectrolytes by quaternary ammonium groups:


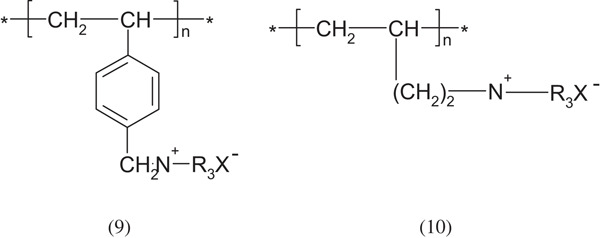


Polymers containing the secondary and tertiary amine groups are clearly less basic, for example poly(4-vinylpyridine), poly(ethylene imine)—examples below. Of course tertiary polymers can be easily quaternized:


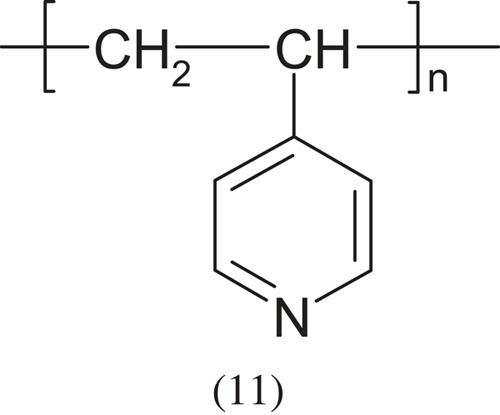


### 1.3. Other structural differences of polyelectrolytes

Due to the high molar mass, the linear shape of the molecules, and the presence of a charge, polyelectrolytes take the form of loose clumps in aqueous or open chains [1,2]. They are used in mining and agriculture. The insolubility of polyelectrolytes can be achieved by the cross-linking process.

Among the anionic and cationic polyelectrolytes, there are strong polyelectrolytes dissociated in the entire pH range (e.g. PSS) and weak polyelectrolytes dissociated in a limited pH range (e.g. poly(acrylic acid), poly(ethylene imine), chitosan).

The physico-chemical properties of polyelectrolyte solutions differ significantly from both low molecular weight electrolyte solutions and non-ionic polymers. The properties of polyelectrolytes in solutions are complex and are a combination of properties resulting both from their macromolecular nature and from interactions between charges. Despite significant progress in the research of solutions of polyelectrolytes (experimental and theoretical), there is still no molecular interpretation of a number of their properties. The physico-chemical properties of polyelectrolyte solutions are determined by the presence of a high ionic charge. The polyion interacts electrostatically with the counterions, forming a molecular system of polyion-counterion with properties different from those of the polyion itself. Electrostatic interactions (Coulomb repulsion of polyions) are responsible for the uptake by chains of flexible polyelectrolytes in aqueous solutions or with a small amount of salt in the stretched, rod conformation. In solutions with a high salt content, the charges are screened and the polyelectrolyte chain takes on a random coil conformation, characteristic of non-ionic polymers.

The chain conformation of many polyelectrolytes, including hydrophobic polyacids in particular, is largely dependent on the pH of the solution. Hydrophobic polyacids show characteristic conformation changes with a change in the degree of dissociation (*α*) as the pH of the solution changes. At low pH values, macromolecules are highly concentrated. With an increase in the pH of the solution and a simultaneous increase in the degree of dissociation, they show a conformational transition from a highly folded to a loosely folded state. This type of conformational transition shows, inter alia, poly(methacrylic acid) with a degree of dissociation of 0.15 < *α* < 0.30 [3].

The electrostatic interactions of the polyion-counterion are the cause of the inhomogeneous distribution of the counterions in the solution. The counterions are mainly located in the immediate vicinity of the polyion. Explaining the transport properties of polyelectrolyte solutions, such as counterion transfer numbers, diffusion coefficients, electrophoretic mobility of polyions, electrolytic conductivity of solutions and sedimentation, and thermodynamic properties, such as counterion activity coefficients, osmotic pressure, and a number of other phenomena in polyelectrolyte solutions, is possible only with the assumption that some of the counterions are ‘bound’ to the polyion, while the remaining counterions are ‘free' [4,5]. The concept of ‘bound’ or ‘associated’ counterions is similar to the definition of ‘condensed’ counterions as found in the Osawa and Manning condensation theory [6].

Regular polyelectrolytes without the addition of salt have a rod conformation in an aqueous solution due to the electrostatic repulsion between homonymous charges placed on the sides of the main chain.

The behaviour of polyelectrolytes in solution depends on both the structure of the polymer itself and external conditions. From the perspective of the compound's structure, attention should be paid to factors such as the type of main chain and side groups, as well as the degree of polymerization. Significant external factors include pH, concentration, the presence of counterions, and temperature. pH, in particular, is considered a crucial parameter as its variations can lead to the polymer being in a coiled state or, due to electrostatic repulsion forces, result in chain expansion and increased aggregate dimensions. Accompanying these effects are characteristic phenomena (concentration effects) manifested through changes in viscosity, sedimentation, diffusion, or light scattering.

A change in the shape of a macromolecule with the same molecular weight leads to a violation of the Staudinger's rule for polyelectrolytes. This deviation provides insights into the shape of the macromolecules present in solution. Studies on the viscosity of polyelectrolyte solutions as a function of various variables allow for determining the viscosity dependence not only on concentration but also on the addition of other electrolytes and the degree of dissociation.

Poly(N,N-dimethylaminoethyl methacrylate) (PDMAEMA) is a relatively new polymer that has attracted the interest of scientists for several years. In its regular form, it is slightly alkaline, and its properties (thermal sensitivity, conformation of macromolecules) largely depend on the parameters of the solvent—pH or type of counterion. The average molecular weight of the polymer and the architecture of the macromolecules also play an important role.

## 2. Polymerization of N,N-dimethylaminoethyl methacrylate

Chain-growth polymerization reactions are reactions in which a polymer macromolecule is formed in several steps. The first is the kinetic chain initiation reaction, the second is the chain growth (propagation) reaction, and the third is the polymer and kinetic chain termination reaction. For each elemental growth reaction, the length of the reactive polymer macroradical increases by one mer. Chain start, growth and termination reactions differ in both the mechanism and speed. Even with a low degree of conversion, the reaction system includes, in addition to the monomer, already formed polymer chains with a high degree of polymerization.

The determination of the molecular weight and shape of polyeletrolyte macromolecules such as PDMAEMA presents many problems due to possible intra- and intermolecular interactions. This is especially true for polyelectrolytes due to the presence of two opposing factors: hydrophobic interactions and Coulomb repulsion. The first enhances inter- and intramolecular interactions and reduces the hydrodynamic volume, while electrostatic repulsion increases the volume of macromolecular clusters. The addition of salt counterions can screen the electrostatic factor but enhances the hydrophobic interactions. This factor can be eliminated by adding surfactants or organic solvents.

Poly(N,N-dimethylaminoethyl methacrylate) is a polymer obtained from N,N-dimethylaminoethyl methacrylate most often by conventional free radical polymerization. However, it should be remembered that it is difficult to polymerize. A dozen techniques have been developed in the literature to obtain polymers with different degrees of polymerization and different polydispersities.

The most commonly used initiator is azobisisobutyronitrile (AIBN), which initiates the process by thermal decomposition:


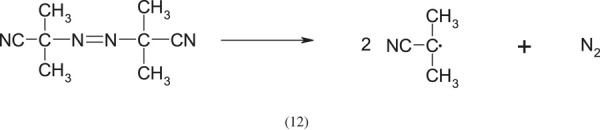


For example, Feng & Huang [7] synthesized PDMAEMA in a solution and prepared the polymer by dissolving the monomer and AIBN in a water-ethanol mixture at 75°C.

The less popular initiators include the bis[2-(bromoisobuturyloxy) ethyl] disulfide ((BiBOE)_2_S_2_) initiator used in the work in [8]. The scheme of its synthesis is shown below:


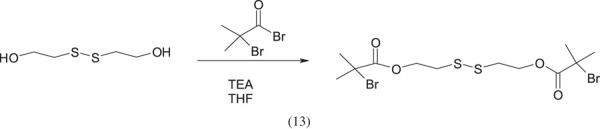


As a result of the use of (BiBOE)_2_S_2_ as an initiator, a polymer with specific end groups is obtained:


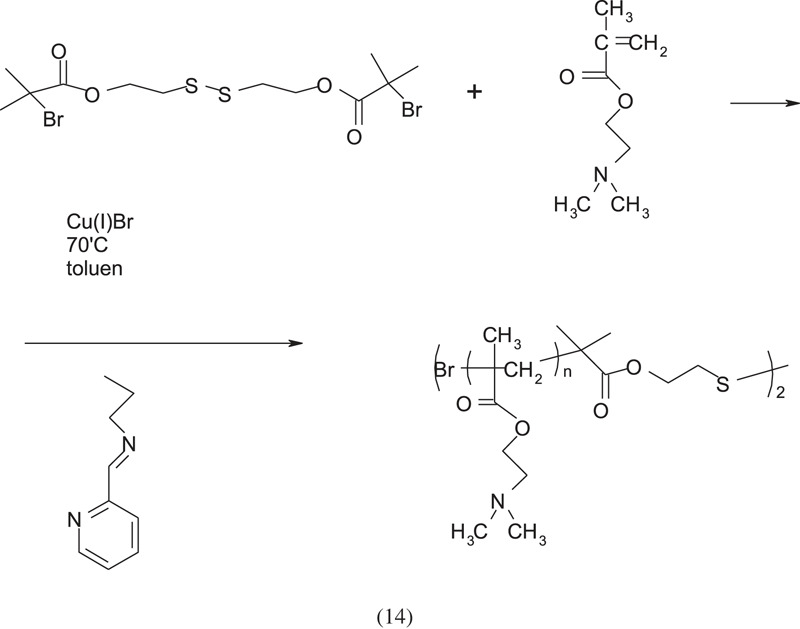


On the other hand, Huang *et al.* [9] used 2-benzophenonyl bromoisobutyrte (BPBriBu), which is a chemically activated initiator, to initiate graft polymerization:


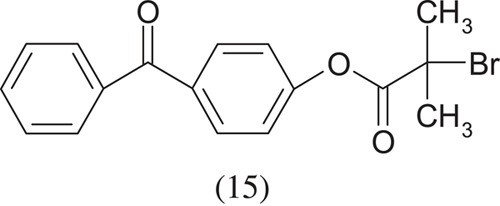


The work of You *et al.* [10] shows the synthesis of PDMAEMA terminated with end groups with sulfide bridges and benzene rings between homopolymer blocks using the reversible addition-fragmentation chain transfer method (RAFT):


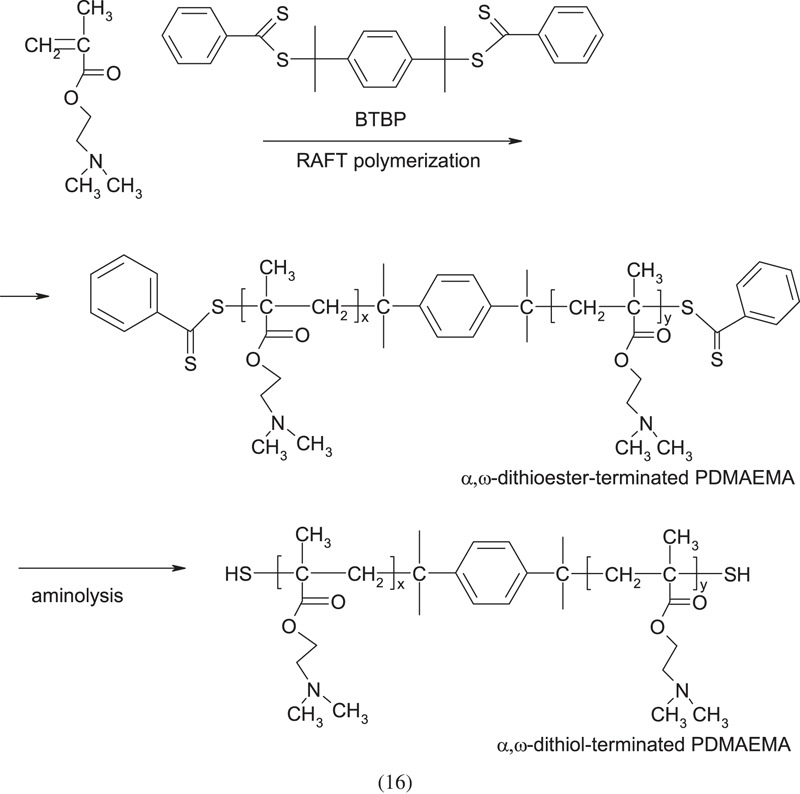


The characteristic parameters of the most commonly used polymerization methods are presented below (table 1).

**Table 1.** Methods of obtaining linear poly(N,N-dimethylaminoethyl methacrylate).

method/reference	solvent	initiator	temp. [^o^C]	time [h]	*M*_n_	*M*_w_/*M*_n_
[11]	tetrahydrofuran (THF)	AIBN	65	24	113 000	1.21
[11]	water	AIBN	not given	not given	184 000,	1.51
					191 000	
[12]	no solvent	2,2-dimethoxy-2-phenyl acetophenone (photoinitiator)	not given	30 min of radiation	not given	not given
[13]	water	ammonium persulfate	60	24	not given	not given
[14]	benzene	AIBN	60		143 000	not given
					151 000	
					196 000	
	toluene	AIBN	60		337 000	
	izopropanol	AIBN	60		335 000	
	water	ammonium persulfate/N,N,N’,N’-tetramethylethylene diamine	25		437 000	
[15]	ethanol	ATRP	not given	not given	not given	not given
[8]	toluene	(BiBOE)_2_S_2_	70	> 15 min	not given	not given
[9]	no solvent	BPBriBu	not given	not given	1500–35 100	not given
[16–20]	xylene	AIBN	70	not given	63 000	not given
[21]	DMF, isopropyl alcohol, ethanol	AIBN	60	24	9000–150 000	not given
[22]	THF	AIBN	60	24	25 000	1.72
		AIBN			87 000	1.29
		AIBN			158 000	1.36
		ammonium persulfate (APS)			326 000	1.62
		APS			408 000	2.26
[10]	THF	AIBN	60	48	M_w_ = 1.8–11.7 × 10^8^	not given
[23–25]	1 M HCl in water	ammonium peroxodisulfate	60	22	131 000	6.24
		AIBN	60	22	20 000	3.15
	toluene				75 000	4.12
[26]	toluene	AIBN	60	24	28 500	1.60
					58 900	1.75
					344 800	1.75
[27,28]	water	ammonium peroxydisulfate	60	24	45 000	8.00
					47 000	5.96
[29]	xylene	tert-butyl peroxy-2-ethylhexanoate	90	not given	not given	not given
[30]	THF	diphenylmethyl lithium	−78	not given	not given	not given
[7]	water/ethanol	AIBN	not given	not given	not given	not given
[31]	no solvent	—	55 (vacuum)	24	not given	not given
[32]	water	ATRP technique, activators regenerated by election transfer (ARGET ATRP)	80	2	not given	not given
[33]	butanone	AIBN	70	8	36 000	2.25
[34]	water	APS polymerization of quaternary monomeric derivatives	50	6	not given	not given
[35]	toluene	AIBN	75 (nitrogen)	10	25500	not given
[36]	1,4-dioxane	AIBN	80, (nitrogen)	3	*M*_w_ = 13 000	not given

PDMAEMA can be synthesized not only by radicals but also by anionic polymerization. Creutz *et al*. [30] investigated polymerization by living anionic polymerization in THF at 78°C using diphenylmethyl lithium as an initiator. That type of polymerization requires rigorous reaction conditions, so it is not preferable in comparison to the free radical process. Another interesting approach to the DMAEMA polymerization process is the modification of the monomer in advance with the low amount of hydroquinone as an inhibitor to prevent an uncontrolled increase in the molecular weight.

Toluene [24] or tetrahydrofuran [7] is used as the solvent quite often. It should be noted that at the presence of the solvent in the polymerization process, the purity of the final polymer product can be poor when the solvent is not removed completely. From that point of view, it is essential to remove the residual solvent before the polymer is used.

### 2.1. Grafted copolymerization of dimethylaminoethyl methacrylate

Classical graft copolymerization is a process involving the formation of branched polymers where the main chain and the branch are macromolecules with a different chemical structure. An advantage of this type of process is the fact that the polymer grafted in this way is permanently attached by a covalent bond. This method of forming copolymers has particular advantages when it is carried out on the surface of another polymeric object and not in its entire volume; a material is obtained that maintains the strength properties of the basic object by changing the structure of its surface layer. In this way, it is possible to introduce thin layers (in classical grafting single layer) with the desired surface. The grafting process, however, is a difficult procedure, requiring the simultaneous creation of special conditions in the reaction environment to allow for the creation of active centres on the modified material as well as conditions for the implementation of the classic polymerization process. It is, therefore, also possible to attach PDMAEMA chains by graft polymerization.

In a previous paper by Madrid *et al*. [37], the authors grafted polypropylene (PP) and polyethylene (PE) nonwovens by the combination of radiation-induced initiation and the RAFT polymerization technique by using 2-cyanoprop-2-yldithiobenzoate as the RAFT agent. The PE and PP graft PDMAEMA with quaternary ammonium functional groups exhibit activity against gram-negative *bacillus E. coli*. This effect is dependent on the absorbed dose, solvent, and monomer concentration. It is also possible to graft cellulose nanocrystals and whiskers by PDMAEMA [38]. The effect of such a modification was obtaining thermoresponsive structures (brush like) that exhibit a chiral nematic structure in the lyotropic state. The aim of that modification was to create temperature-induced structures that could use the lower critical solution temperature of PDMAEMA macromolecules. Another example of grafting PDAEMA macromolecules to a modified object is a paper in which the authors presented grafting to a polyamidoamine dendrimer, which is biocompatible, nonimmunogenic and possesses terminal-modifiable amine functional groups for binding various targeting or guest molecules [39]. The authors used chlorambucil as a model drug and found that the controlled drug release from the dendrimer derivative can be effectively controlled by the pH value of its environment. Another paper by Fang *et al.* [40] reported grafting of DMAEMA via the ATRP process utilizing the labile chlorines on PVC backbones. The authors obtained a hydrophilic membrane for nanofiltration. Also, the ATRP method was used to graft such chains onto poly(ether ether ketone) [41].

Another example of using the DMAEMA grafting technique to impart the properties of this polymer to other materials is the attachment to gold nanoparticles (AuNPs) [42]. The reaction conditions were set so as to obtain different lengths of attached polymer chains. After confirming the grafting process with basic techniques (UV-visible, FE-SEM, AFM, TEM, FT-IR and TGA), the stimuli responsive behaviour of the PDMAEMA-grafted AuNPs was tested. It has been shown that at a low pH and temperature, the obtained nanoparticles become well dispersed over the entire range of molecular weights. However, the authors showed that the molecular weight of the grafted polymers becomes important at a neutral or alkaline pH.

Another interesting example of the creation of graft copolymers is the grafting of PDMAEMA on poly(vinylidene fluoride) [43]:


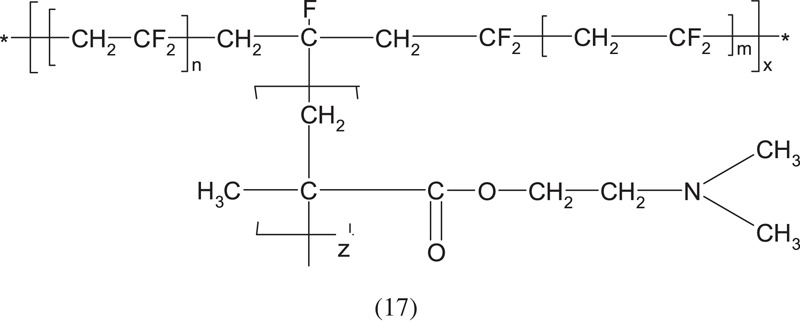


The authors found that the graft length is independent of the time of polymerization but the graft density depends on the polymerization time.

### 2.2. Star shape polymers and brushes

PDMAEMA brushes were prepared by Lee *et al*. in [44]. They copolymerized a small amount of a light sensitive azobenzene monomer to investigate the observed solubility characteristic points as a function of lighting. The brushes with cisazobenzene units did not show any macroscopic demixing, where the brush with trans-azobenzene units presented a moderate reduction in transmission at a rather high concentration (1 wt%).

Georgiou *et al.* [45] investigated the thermoresponsive properties of PDMAEMA stars, which were prepared by the arm-first method. They have large hydrophobic cores. The authors also reported that the solubility point in pure water does not depend strongly on the arm number (a difference between 29°C for a star with 24 arms and 34°C for a star with 50 arms).

A previous paper [46] reported the investigation of the thermoresponsive properties of a well-defined set of star-shaped PDMAEMA. The polymers were prepared by the core-first method, and stars with up to 24 arms and a low polydispersity (1.43) were obtained. By contrast, PDMAEMA stars made by the arm-first method usually exhibit higher polydispersities (e.g. >1.6), have a slightly large hydrophobic core, and possess hydrophobic initiator moieties attached at the periphery.

A star-shaped polymer comprising a cyclodextrin core and PDMAEMA arms was shown in [47]. This star vector was shown to condense DNA into nanocomplexes.

In [48], the synthesis of star PDMAEMA via the ATRP method using the arm-first method was shown. The core was N,N,N′,N′,N″-pentamethyldiethylenetriamine (below).


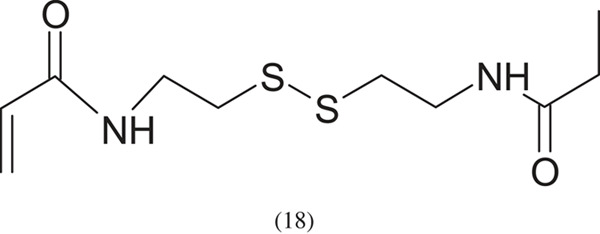


PDMAEMA stars and their quaternized analogues were successfully synthesized by the core-first method employing ATRP [49]. The structure of the initiating system together with the polymerization features lead to polymer stars with an adequate arm number (up to 24) and arm size distribution due to the slow initiation. Quaternization changes these weak electrolytes into well-characterized strong polyelectrolyte stars.

An interesting possibility is to make modifications of DMAEMA before it is polymerized. For example, in [50], the monomer is modified to a quaternary ammonium salt form using HCl, H_2_SO_4_, HNO_3_ and H_3_PO_4_. The authors then perform block polymerization. The process is controlled by the DSC technique, including its kinetic parameters.

## 3. Properties of poly(N,N-dimethylaminoethyl methacrylate)

The properties of PDMAEMA depend on many polymer parameters, such as the average molecular weight, polydispersity, presence of impurities, and possibly the degree of cross-linking. The researcher can control most of these parameters by conducting the polymerization process appropriately.

### 3.1. Solubility

PDMAEMA is a linear macromolecule and is soluble in aqueous solutions. During bulk polymerization, DMAEMA can be automatically cross-linked by chain transfer to the polymer. However, the auto-crosslinking is too weak to prevent PDMAEMA from dissolving in water. PDMAEMA has a structure similar to DNA [24]. This polymer has a p*K*a close to 7.9 in water (close to the physiological pH) [51], is a weak polyelectrolyte, and shows a lower critical solution temperature (LCST). Depending on the conditions (average molecular weight, pH, type of salt and salt concentration), values of the LCST vary in the range of 38–50°C in aqueous media.

Thermoresponsive polymers, which show a pronounced change in their solvation at a certain temperature, have attracted much attention in current research. Thermoresponsive polymers, which are water-soluble as PDMAEMA, are of particular interest for applications under physiological conditions. The upper solubility points reported in literature vary from 14°C to 50°C in pure water [52–57]. This gives some indication of a class I LCST behaviour (LCST depends on molecular weight).

The introduction of charges leads to a further, effective stabilization of macromolecules in solution against phase separation and aggregation. PDMAEMA is a suitable polymer to analyze the effects of charges on the LCST because it is a weak cationic polyelectrolyte. The thermoresponsive properties of the polybase PDMAEMA can then be controlled by small changes in the pH and salinity as well.

The properties of this polyelectrolyte can be significantly changed by using it in a protonated form. Hunley *et al*. [11] modified PDMAEMA obtained by method 1 (table 1) using HCl, HBF_4_, and CF_3_SO_3_H as a titrant, titrating the polymer to pH = 5 and obtaining a polymer in the form of PDMAEMA•HCl, PDMAEMA•HBF_4_, and PDMAEMA• HOTf, respectively. Then, further ions were introduced by ion exchange:


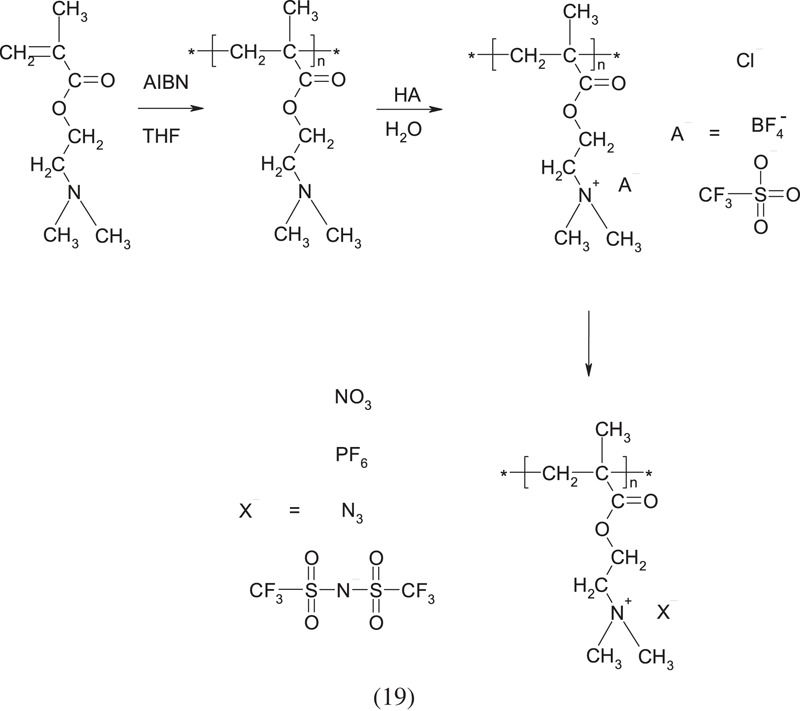


The introduction of various types of counterions makes it possible to obtain a polymer with altered solubility, e.g. some of the derivatives, in contrast to non-ionizing PDMAEMA, are insoluble in water or acetone [11].

### 3.2. Dissociation constant and conductivity

The apparent dissociation constant in water shown in [26] is between 6.5 and 7 and is rather independent of its degree of ionization. It can be assigned to cyclic conformations of ionized and non-ionized tertiary aminoalkyl esters.

Water-soluble polyelectrolytes are very dependent on the presence and type of counterion. In an aqueous solution, the Cl- anion is mostly dissociated and gives good conductivity [11]. The conductivity in the solution may be ranked (at constant molar ratio) according to the type of counterion as follows: Cl- > PF_6_- > BF_4_- > N(CN)_2_- > TfO-. The decrease in conductivity is closely correlated with the decrease in the mobility of the counterions [58]. In dilute solutions, the mobility of the small counterions is dominant compared to the large polycation.

In a prior work [31], it was found that both PDMAEMA in its basic form and in the hydrochloride form show ionic conductivity. It was found that the polymer in both forms exhibits conductivity in a wide range, frequency, and at different temperatures.

### 3.3. Thermal properties

The results of the thermogravimetric analysis shown in [11] give the 50% decomposition temperature (T50%) of PDMAEMA (obtained by method 1, table 1) at about 380°C. The decomposition has a two-stage character; the first stage is related to the degradation of the side groups and the second is related to the degradation of the main chain. The addition of counterions can significantly reduce the thermal resistance (for example, for PDMAEMA•HCl, T50% is about 275°C) or improve it (for PDMAEMA•HOTf, T50% = 415°C) [11]. In the latter case, the degradation process has a single-stage character.

The most in-depth studies of the thermal properties of PDAMEMA were published in [59]. In order to obtain complete information on the course of thermal decomposition, the sample was heated in various temperature ranges. The samples obtained after the assumed heating time were examined using infrared analysis. The two-stage process of thermal decomposition was confirmed based on the thermogravimetric curve. The limiting temperature for the first stage was about 390 ^o^C, after which the second stage of sample destruction started. It was also confirmed that the disintegration of side groups takes place in the first phase of thermal degradation and the combined processes of disintegration of side groups and the main chain occur in the second stage. Based on the infrared spectra, it was found that with the increase in the amount of thermal energy supplied, the intensity and proportions of signals in the FTIR spectra changed. Additionally, new signals emerged. It was confirmed that in the first phase of the first stage of decomposition, the amino groups undergo thermal destruction, and in the second phase, the disappearance of side groups can be observed. The second stage of thermal decomposition of PDAEMA took place in the temperature range of 390–560°C. After identifying the FTIR spectra, it was found that the degradation of the side groups of the tested polymer continued and the degradation of the main polymer chain started.

The activation energy values of the thermal decomposition process were also determined; they are 89.8 kJ mol^−1^ for the first stage and 17.7 kJ mol^−1^ for the second stage of the degradation process.

### 3.4. Hydrodynamic parameters

Comprehensive research on PDMAEMA macromolecules in solution can be found in [14]. The determined diffusion coefficients of the non-ionized polymer (0.001 M NaOH in methanol) range from 1.67 **×** 10^−7^ to 10.0 **×** 10^−7^ cm^2^ s^−1^ for samples obtained under different conditions (measurement conditions: *T* = 24.6°C, solvent density 0.8 g cm^−3^, solvent viscosity 0.6 **×** 10^−2^ P). The refractive index increment dn/dc is 0.17, and the values of the sedimentation coefficient(s) extrapolated to zero concentration are from 1.1 **×** 10^−13^ to 9.3 **×** 10^−13^ Sv.

### 3.5. Antimicrobial properties

The growing interest in a broadly understood healthy lifestyle in modern society also causes an increase in the demand for materials with antibacterial properties. Thanks to these types of materials, an environment free from pathogenic microorganisms is created in our immediate environment. It should be remembered that natural products valued for the comfort of use, such as cotton, create an ideal environment for the growth of bacteria in contact with the human body. Attacks of microbes pose a threat to humans both when bacteria or fungi are alive and dead. The spread of diseases, the emission of an unpleasant odour, and faster destruction of the material are some additional effects of the attack of bad microbes.

Additionally, pollution caused by microbial adhesion and proliferation on synthetic surfaces such as food processing, packaging, food service, hospital equipment and the waste-water treatment industries, as well as individual households, is a major concern. Patients are significantly threatened by different types of hospital infections, which present a serious challenge. Therefore, the development of new antibacterial materials is always needed.

The antibacterial properties of tertiary PDMAEMA have been thoroughly investigated and described by Rawlinson in [8]. A series of analyses of the action of the polymer on gram-positive and gram-negative bacteria were carried out. PDMAEMA inhibits the growth of all the negative bacteria tested and shows variable effects in relation to the positive ones, and no inhibitory effect on yeasts was demonstrated. These results were obtained after an 18-hour incubation period of the bacteria. In the works described in [9,60], the action of PDMAEMA against *Escherischia Coli* was also investigated. The high efficiency of the tested polymer was found. Rawlinson *et al.* [8] explain the difference with different incubation times for bacteria. In [9,60], the bacteria were incubated for 1 h, and after using longer periods of time (e.g. 18 h), the bacteria can start the process of regrowth. On the other hand, in the work described in [61], the effectiveness against *Escherischia Coli* was also found after 30 min of incubation.

The mechanism of antimicrobial activity of tertiary nitrogen in PDMAEMA probably depends on the hydrophilic–hydrophobic balance in the macromolecule. The main hydrophobic chain provides compatibility with the lipid bilayer of the bacterial cytoplasmic membrane, and the hydrophilic side groups offer solubility in water solutions [62]. Rawlinson *et al*. [8] describe the mechanism of PDMAEMA's antibacterial action as follows:

— the initial step in the mode of action is adsorption onto the bacterial cell surface,
— the second step is the diffusion through the cell wall and binding to the cytoplasmic membrane. The variation in the antibacterial effect between gram-positive and gram-negative bacteria may be partially explained in this step.
— the final steps in this mechanism are the disruption of the cytoplasmic membrane, release of cytoplasmic constituents, and cell death.

The other theory describing the mechanism of antibacterial activity of quaternary ammonium salts believes that polycations are able to replace the divalent cations of the bacterial cellular membrane, which are responsible for the neutralization of the membrane, leading to the loss of membrane integrity [63,64].

Polylactide nonwovens padded with a 1% PDMAEMA solution showed biocidal activity against the bacteria *Escherichia coli, Bacillus subtilis*, and *Staphylococcus aureus* [19]. The surfacing technique is also described in [20]. The half-percent solutions were introduced alternately with the silver salts of the poly(acrylic acid). The authors expected a cumulative biocidal effect of PDMAEMA and silver. It turned out that samples with PDMAEMA outer layers showed excellent killing properties against *Staphylococcus aureus* bacteria, i.e. 100% of the microorganisms were killed. Interestingly, nonwovens with outer layers of silver salts practically did not have a bactericidal effect.

The fibres were also investigated in [32]. The silk fibres were grafted with PDAMEMA to obtain the stable binding of the modifier and the killing activity against *E. Coli* and *S. Aureus* bacteria.

Another study by Lee-Anne Rawlinson [65] compared the biocidal activity of PDMAEMA against *Staphylococcus epidermidis* and *Staphylococcus aureus*. The tested polymer was shown to be more effective against *S. epidermidis*, further inactivating films from mature bacterial strains. PDMAEMA as an amphiphilic polymer contains both hydrophobic and hydrophilic regions. The hydrophobic part may be important in the permeabilization of bacterial membranes [65]. The hydrophilic fragments are implicated in enabling diffusion of antimicrobial agents through the polysaccharide matrix of biofilms and in the initial attraction of the bacteriostatic agent.

As known from the literature [66], quaternized materials can act as cationic antibacterial agents to kill bacterial cells. The mechanism of this process consists of the following steps: (a) the attachment of a quaternized substance to the surface of a bacterial cell, (b) adsorption onto the cytoplasmic membrane through the cell wall, (c) disruption of the cytoplasmic membrane, and (d) leakage of the cytoplasmic constituents. As a result, the quaternized substance causes irreversible cell death. The antibacterial activity of positively charged quaternary ammonium salts is due to an interaction with the negative cell surface.

Quaternary ammonium compounds, mainly salts and hydroxides, are finding more and more use in various industries due to their many useful properties. In 1890, the Russian chemist Nikolai Menschutkin synthesized them for the first time, and the amine quaternization reaction (Menschutkin reaction) is often called by his name. Already in the 1930s, ammonium compounds were readily used ingredients in cleaning and disinfecting preparations. Due to their high thermal stability, odourlessness, colourlessness and a relatively wide spectrum of activity, quaternary ammonium bases have dominated the market of available disinfectants for over 20 years [67]. Quaternary ammonium compounds are ionic compounds that contain 4 organic groups in the molecule linked to the nitrogen atom (including 3 covalent bonds and 1 coordination). The order of the amines is determined by the number of hydrogen atoms and the lone pair of nitrogen electrons that are substituted with carbon atoms in the ammonia molecule. They are amphiphilic in nature, i.e. the hydrophilic element of the molecule is the nitrogen cation and the hydrophobic part is the alkyl chain. Such a chemical structure provides unique activation properties at the interface and interesting possibilities of surface interactions. The surface activity of quaternary ammonium compounds is also determined by the length of the aliphatic carbon chain—it can have up to 14 carbon atoms. In aqueous solutions, ammonium compounds undergo electrolytic dissociation and lower the surface and interfacial tension. They have been used in many application processes. Antimicrobial activity is related to the adsorption of ammonium cations by the negatively charged cell wall and the subsequent penetration of the compound into the cytoplasm. This causes permanent damage to the wall, disrupting the mechanisms of maintaining ion balance, and consequently leads to the inhibition of metabolism and even cell death. The lower activity of quaternary ammonium compounds against gram-negative bacteria is due to the different structure of the cell wall of these bacteria compared to gram-positive bacteria and is associated with the presence of smaller diameter channels, which hinder the penetration of chemicals from the external environment into the cell [68,69].

The PDAEMA polymer contains a tertiary amino group on the side chain, which has a relatively high reactivity and can be quaternized to form a quaternary ammonium salt:


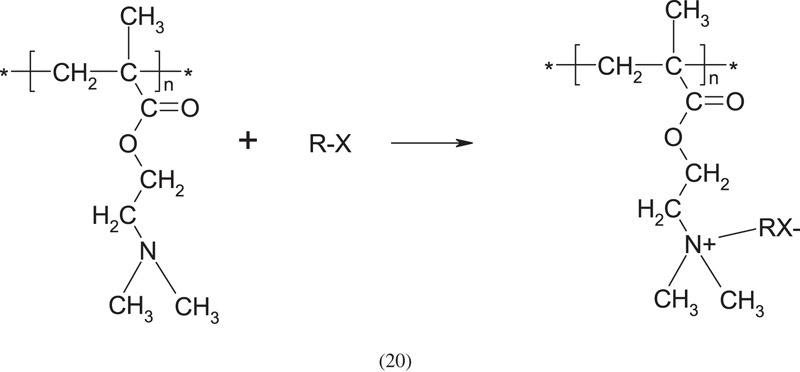


where: R maybe an alkyl or phenyl monohalide, X- the negative mobile counterion.

Using the amino group as a nucleophilic reagent, the halogen atom is replaced by the amino group, finally forming a quaternary ammonium salt. Treatment of tertiary PDAEMA with such mono-halohydrocarbons as CH_3_I and C_2_H_5_Br, according to the above scheme, leads to water-soluble products. The quaternization reaction can be easily used to crosslink PDMAEMA by using bifunctional reagents, for instance, alkyl dihalide. Difunctional halohydrocarbons (e.g. 1,4-dibromobutane) can be used for the quaternization reaction with two tertiary amino groups from the same or different macromolecules. When the quaternization takes place on two different macromolecules, it crosslinks the polymer. An example of the reaction between two amino groups on PDMAEMA and the binary halohydrocarbon is as follows:


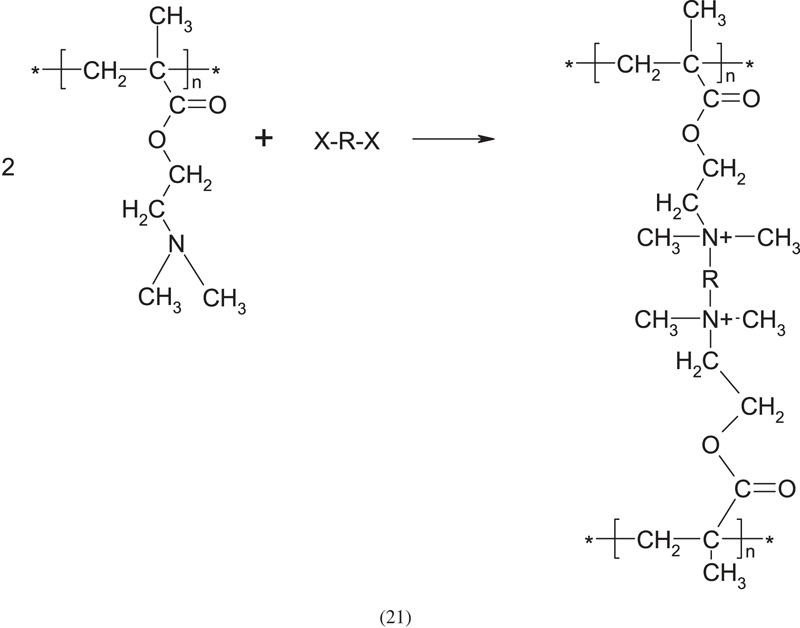


A quaternary PDMAEMA derivative was used in a prior work by Wang *et al.* [15] as an outer layer seeded on silicone, and 95% of bacteria were killed after 18 h of exposure to the silicone nanowires.

The authors [50] investigated the activity of a polypropylene with surface grafted quaternary PDMAEMA chains against *E. coli*. A very high antibacterial effectiveness of the tested samples was found. A material with grafted macromolecules with *M*_n_ over 10 000 killed 100% of the bacteria. A correlation was observed between the antibacterial effectiveness and the average molecular weight; after inoculation of the polymer with the lowest *M*_n_ (greater than 1500), the effectiveness was reduced to 85%.

Obviously, the effectiveness of bioactive quaternary derivative interactions significantly depends on the degree of quaternization, i.e. the ratio between groups with tertiary nitrogen and quaternary ammonium salt. In [70], Barboiu *et al.* demonstrated the possibility of using the ^1^H-NMR technique to determine the proportion of nitrogen in the tertiary and quaternary forms.

However, it should be remembered that the widespread use of quaternary ammonium compounds as antibacterial agents carries the risk of developing allergic diseases and those resulting from the irritation of these chemicals [71]. The potential effects of exposure to such compounds should not be underestimated—they should be used with caution and in compliance with all hygiene standards.

It is also known that quaternary PDMAEMA alkylated by alkyl halides can be used as a DNA co-complexing agent [72], and it works more effectively in this respect than the tertiary polymer. For example, the paper [35] presents a process in which PDMAEMA was reacted with alkyl halides, benzyl chloride, propyl bromide, 2-methylpropyl bromide and 3-methylbutyl bromide. As a result, quaternary derivatives were obtained. The authors confirm that all modifications were made only on the nitrogen atom and there was no double bond.

Some studies also suggest that PDMAEMA has anti-cancer properties. For example, the authors of [73] provide such information by citing sources [8,61]. However, a careful analysis of the above sources did not confirm the above suggestion.

### 3.6. Cytotoxicity

Generally, polycationic molecules are cytotoxic [48]; however, the dose required to kill cells depends on the nature of the polycation. To use PDMAEMA as an antimicrobial agent, it must be shown that it is safe to humans. Reports on the cytotoxicity of this polymer vary depending on the method of administration, possible quaternization, cell type and conditions [74–77].

In [74], it was shown that PDMAEMA caused little or no hemolysis to human red blood cells when injected intravenously into the tail veins of rats. It caused death at 5.1 mg kg^−1^ but was tolerated at 2.1 mg kg^−1^.

Yancheva *et al.* [76] showed that PDMAEMA did not cause hemolysis of red blood cells; it did encourage hemagglutination. Polyelectrolyte complexes between quaternized PDMAEMA and crosslinked chitosan derivatives (N-carboxyethylchitosan) or poly(2-acrylamido-2-methylpropane sodium sulfonate) (PAMPSNa) revealed that the complexes had lost the inherent PDMAEMA cytotoxicity but still preserved haemostatic activity. On the other hand, the complex between quaternized PDMAEMA and PAMPSNa had improved the blood compatibility [76].

In the work in [8], Rawlinson *et al.* investigated the cytotoxicity of the polymer in relation to sheep erythrocytes. Up to a concentration of 10 mg cm^−3^, no significant hemolysis effect was found. Additionally, the cytotoxic activity against a human intestinal epithelial cell line and human monocytic cell line was investigated.

It has been previously shown that PDMAEMA has a similar cytotoxicity to the natural polymer chitosan, which is considered nontoxic [22], when added to human epithelial cell layers. PDMAEMA prevents disruption of the cells after treatment with bacterial toxins.

On the other hand, Cherng *et al.* [27] showed the cytotoxic effect of PDMAEMA used for the purpose of gene transfer, as well as already formed complexes with DNA [24]. PDMAEMA for gene transfer was tested for cytotoxicity against human brain microvascular endothelial cells and 3-[4,5-dimethylthiazol-2-yl]2,5-diphenyltetrazolium bromide (MTT). A cytotoxic interaction was found, with the lower molecular weight polymer samples being somewhat less toxic. However, the concentration of PDMAEMA had a much greater influence on cytotoxicity.

Results shown in [48] by Dai *et al.* demonstrate that when PDMAEMA is incubated with cultured human cells, it is a potent cytotoxin agent. At high concentrations (above a few micrograms per millilitre), PDMAEMA killed most of the human myelomonocytic cells in less than 15 min. At lower concentrations, PDMAEMA was slower acting and killed fewer cells; however, even 2 mg cm^−3^ PDMAEMA destroyed about 50% of the exposed cells within 1 h. Dai *et al.* [48] also found that lowering the temperature from 37°C to 4°C decreased the cytotoxicity of the polymer by about 80%. According to the investigation, the mode of cell death is primarily necrotic in character. In a previous paper [48], the mechanism of cytotoxic activity of PDMAEMA was analysed in detail.

Another previous paper [75] showed that the cytotoxicity of the PDMAEMA alone at *in vitro* conditions depends on its structure in the following order: star-shaped PDMAEMA > linear PDMAEMA = highly branched PDMAEMA. The results presented in [75] demonstrate that when unmodified PDMAEMA is incubated with cultured human cells, it is an effective cytotoxin. At concentrations above a few micrograms per milliliter, PDMAEMA killed most human myelomonocytic cells in less than 15 min. At lower concentrations, the polymer killed fewer cells and acted slower; however, even 2 µg ml^−1^ of PDMAEMA killed about 50% of the exposed cells within 1 h. According to [75], ‘*the mechanism of toxicity involved cells transiting through a stage where their membranes had a permeability to propidium iodide that was intermediate between that of healthy cells (low) and dead cells (high), indicating that their cell membranes had become ‘leaky*’. The recognition of ‘leaky’ cell membranes following exposure to PDMAEMA increases the possibility that the polymer may use toxic action by directly interacting with cell membranes.

PDMAEMA acts destructively against healthy African green monkey cells [78]. The cytotoxicity effect of PDMAEMA was demonstrated by the important decrease in cell viability at a concentration above 50 µg ml^−1^. Like other cationic polymers, PDMAEMA is slightly cytotoxic. The important cytotoxicity of PDMAEMA may limit its application in therapeutic applications. Conclusions about the toxicity of PDMAEMA must be the subject of a deeper analysis.

### 3.7. Sorption and hydrophilic properties

The parameters of hydrophilicity or hydrophobicity of different polymers depend on the purpose they are designed for. The absorption of water by textiles can proceed as a result of its chemical structure and different factors:

— water penetration into the inside part of materials until their maximum hygroscopic saturation;
— wetting the surface of materials and the component fibres;
— wetting the capillaries and slots formed in the material (capillarity);
— pressing water into free fabric spaces under pressure.

Materials subjected to the action of water contain most frequently some moisture derived from their surroundings. Their contact with water brings about outer wetting as well as an increase in their moisture content until the maximum hygroscopic state.

Another way to apply the cationic functional groups in PDMAEMA is to use them for sorption of inversely charged ions from solution. Due to the good solubility of the polymer in its basic form, the quaternization procedure with cross-linking is used. In the work in [79], polyolefin fibres are pre-activated by radiation and then grafted with DMAEMA and a quaternized dimethylsulfate solution in DMF. The fibres obtained in this way were used to effectively remove arsenic in the form of arsenous acid from the water.

An alternative possibility that allows the use of sorption properties is the synthesis of hydrogels. In [80], Ning *et al*. investigated the properties of hydrogels obtained by the radiation method. Optimal synthesis conditions were determined, where the variables are total dose, dose rate and the concentration of the monomer and crosslinker in the aqueous solution.

### 3.8. Complexing interactions

Polymer-metal complexes can offer many valuable properties, for instance, thermal, electrical, mechanical or catalytic. The use of a polymer carrier makes it possible to combine the properties of the macromolecular matrix with the ligands. PDMAEMA contains a tertiary nitrogen with a free electron pair, which allows metal complexes to be effectively created. The structure of the complex depends on several factors: stoichiometry, chemical formula of the ion, temperature, or pH. The work presented in [81] shows the creation of PDMAEMA complexes with FeSO_4_·2H_2_O, CoCl_2_·6H_2_O, CuCl_2_·2H_2_O, VOSO_4_·5H_2_O, Na_2_MoO_4_·2H_2_O and Na_2_WO·2H_2_O.

The use of a positively charged tertiary nitrogen in the PDMAEMA macromolecule as a complexing agent allows new chemical structures to be obtained. These are insoluble systems with new functional groups derived from the second reactant. For example, in the work in [78] is described a complex of PDMAEMA with chondroitin sulfate at different pH values. It turned out that in the complexed form, the system has a lower cytotoxicity (against African green monkey cells) than the base polymer. This may suggest the development of biomaterials based on the PDAEMA polymer.

## 4. Practical solutions allowing the use of

### 4.1. Poly(N,N-dimethylaminoethyl methacrylate)

The excellent solubility of PDMAEMA creates many opportunities to use it in various technological applications to obtain a product containing PDMAEMA in its structure. Textile applications constitute an important group of applications of the described polymer.

### 4.2. Thin layers created by the grafting method

The grafting of the PDMAEMA macromolecule to the outer layer of another polymer makes it possible to obtain a material with the bulk properties of the main, more common, and inexpensive polymer and with changed surface properties. PDMAEMA was introduced onto the surface of silicon nanowire arrays by graft polymerization using the ATRP technique [15]. In the next step, the polymer was quaternized with benzyl chloride. As a result of this process, nanowires were obtained, showing antibacterial properties against *Escherichia coli*. Comparing the effects of the modification of silicone nanowires with silicone foil, the authors found that a much higher cross-linking density was obtained on the nanowires. As a result, the adhesion of bacteria to the hydrophilic (after modification) surface of the nanowires was much higher. It allowed a very high level of antibacterial effectiveness of the nanowires to be obtained—95% of bacteria killed after 18 h compared to a similarly modified smooth silicone (45%).

Grafting of PDMAEMA on the polypropylene surface by combining a novel photochemical method with a controlled radical polymerization technique was described in [9]. Surface modification was confirmed by using FTIR reflectance spectroscopy and contact angle measurements. The grafting density varied between 0.19 and 0.23 chains nm^−2^.

On the other hand, in [32,82], PDMAEMA was attached to silk fibres. This type of natural fibre has antibacterial properties that can be enhanced with an additional outer layer. As a result, a more intense and broader action in terms of the microorganisms being combated can be expected. The authors confirm the biocidal activity against *Escherichia coli* and *Staphylococcus aureus* bacteria, and also improvement of the thermal properties. The modification carried out is resistant to washing; however, some deterioration in the mechanical properties is noted.

Another possibility is to use the PDMAEMA macromolecule as an object to which another polymer will be attached (see Scheme below). An example of this type of use of PDMAEMA is ɛ-caprolactone grafting [83]. The authors used a three-step procedure to obtain a polymer with amphiphilic properties:


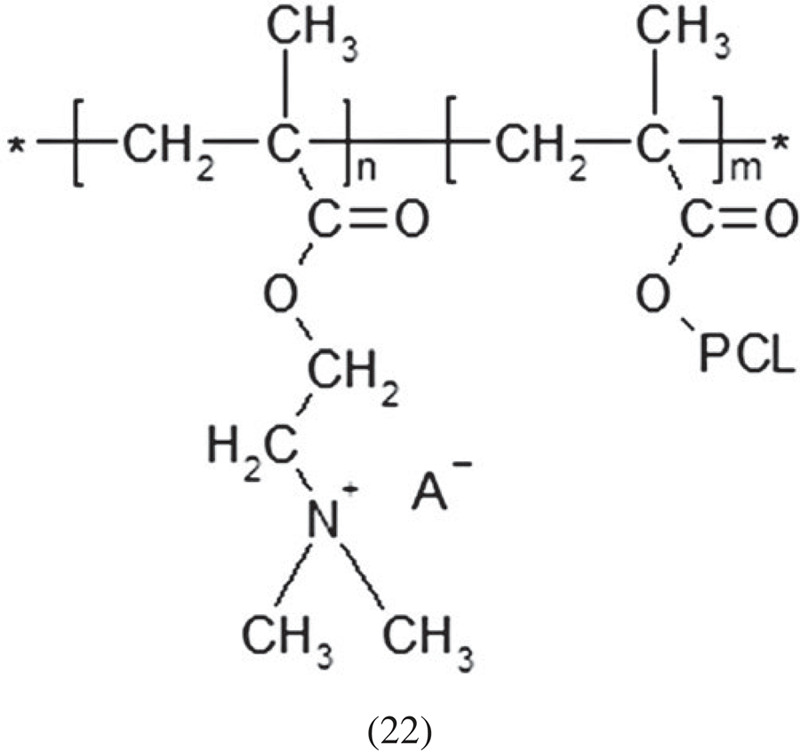


### 4.3. Electrospinning

The scientific literature describes the preparation of fibres from non-ionized PDMAEMA using the electrospinning technique [11,13]. The diameter of the obtained fibres ranged from 285 nm to 4560 nm depending on the concentration [11]. Hunley *et al*. [11] also produced fibres by the PDMAEMA technique of electrospinning with counterions. As expected, solutions containing smaller, mobile counterions allow for the production of fibres with smaller diameters (under the same spinning conditions). The electrospinning technique was also used in [13]. In this case, the HCl counter-ions are already added to the monomer at the polymerization stage. The fibres made of PDMAEMA·HCl were found to have a much smaller diameter than that of the inert polymer. The authors suggest the use of PDMAEMA·HCl in apparel applications as a chemically and biologically active agent, especially due to the presence of a quaternary nitrogen atom.

### 4.4. Padding

The external layer padding technique is one of the simplest methods to introduce the modifier. The material is introduced into a solution of the modifying substance at an appropriate concentration; then, as a result of physical, physico-chemical or chemical interactions, the modifier connects to the surface of the main, basic material or to other layers introduced earlier. The padding method is simple from the application point of view; however, it is often associated with obtaining a weak bond of the modifier to the surface.

Połowiński *et al.* [19] used padding of a polylactide nonwoven with a previously introduced layer of poly(acrylic acid) with a 1% PDMAEMA solution, obtaining as a result of complexing interactions an insoluble surface system. It was found that after the application of PDMAEMA, the hydrophilic parameters deteriorated, but at the same time, the non-woven fabric obtained bactericidal properties.

The padding of a nonwoven fabric, this time polypropylene, is also described in [20]. In this case, a 0.5% solution was used. The layers of PDMAEMA were applied alternately as a positively charged electrolyte and silver ions (negative charge). The surface structure of four layers connected by electrostatic interactions was obtained. Thick macro-layers were introduced, obtaining significant gravimetric effects—the four-layer system increased the mass of the modified sample by 43%. The structure was confirmed by using FTIR spectroscopy (ATR reflectance) or XPS spectroscopy. As one of the possible modification goals was to obtain nonwovens for filtration purposes, air permeability tests were also carried out. It was found that the applied modification scheme did not significantly affect the flow properties. However, samples with PDMAEMA outer layers showed excellent antibacterial properties.

### 4.5. Surface deposition by using a layer-by-layer technique

Preparation of thin, mainly organic layers containing functional groups and changing the surface properties of modified planar objects are currently of wide interest [16,84–94]. Thanks to this type of modification, it is possible to obtain products combining the bulk properties of the main object with the surface parameters of the new outer layer. By appropriately selecting the type of the applied layer, it is possible to precisely control the surface properties of the modification object, adapting them to specific requirements. The main assumption of the layer-by-layer (LbL) method is the application of alternately charged layers of polyelectrolytes that interact with each other due to electrostatic forces. The originator and pioneer of this method was Gero Decher, who published in the 1990s a series of works describing this method [84–89]. Since then, the method has been further developed by many researchers [16,95–98], including Decher himself [99–101].

When discussing the theoretical foundations of the LbL method, it should be remembered that although it is commonly used to associate this name only to systems connected by electrostatic interactions, Decher himself [102] extends the scope of acceptable interactions in this definition range to almost all besides main bonds: hydrogen bonds, donor–acceptor interactions and many more. Decher [102] also shows a far-reaching analogy between the scheme of the standard multistage reaction and the modification of the LbL type with successive stages in the form of successive layering.

The production of foil by the LbL method means the production of complex multicomponent systems in the nano-scale in a very inexpensive and environmentally friendly way. Electrolyte or polyelectrolyte nano-layers can be applied to various types of flat surfaces: mica, glass, quartz, gold, titanium, silicone, pigment particles, polymer films (membranes), microspheres [102] or fibres [103].

The deposition of polyelectrolyte layers on the surface of fibres or textiles using the LbL method was initially described in the scientific literature only in recent years [92,104–112]. In the work in [104], the deposition of layers on the surface of a straightened single regenerated cellulose filament placed on a silicone plate was described. Meanwhile, paper [112] describes the modification of cotton fibres and products. The surface was activated using 2,3-polypropyltrimethylammonium chloride to produce the first positively charged layer (cationization). Alternately, poly(styrene sulfonate) (PSS) and poly(allyl amine hydrochloride) (PAH) were applied. To confirm the deposition process, the authors used the XPS technique, which gives similar results to those obtained for such a system on a different plot. The thickness of one layer, calculated on the basis of TEM images is 16–19 nm. The authors do not investigate changes in the characteristic properties of the surface. Similarly, in the work in [106], the LbL method was also used to apply PSS and PAH to cotton fibre. To confirm the modification, infrared reflection, XPS spectroscopy and TEM microscopy are used. However, in [108], layers of dye are applied alternately with PSS on the natural silk fibre. The surface is analysed with FTIR and UV-VIS spectrophotometric reflection techniques.

The work in [92] describes the deposition of polyelectrolytes on the surface of an actual textile product (non-woven fabric). In it, polypropylene and polyester nonwovens were modified, and the deposition was confirmed using an electron microscope (SEM) and examining the remission of test-stained samples. The conditions of grafting and the dyeability of the modified nonwovens are also described in detail in [113]. PDMAEMA as a positively charged polyelectrolyte has been used as an agent modifying the surface of textiles in several works [16,17,92,103,110,111]. The purpose of introducing PDMAEMA nanolayers on the fibre surface can be different. In the work in [16], PDMAEMA was one of the layers on the surface of the fibres in a polypropylene non-woven fabric—the positive charge of the polymer allowed for the introduction of noble metal nanoparticles (gold, silver, platinum) in the next stage. As a result, a significant improvement in the thermogravimetric characteristics of the material was obtained, shifting the temperature of 50% of decomposition by even about 30°C (70°C after platinum hydrogenation). In the work in [17], nano-layer systems were obtained on polylactide non-woven fabric using the layer-by-layer technique, where PDMAEMA was introduced twice. The first layer of poly(acrylic acid) was grafted onto the surface, followed by the alternating deposition of PAA and PDMAEMA until a total of five layers were obtained (table 2).

**Table 2.** The system of layers deposited on the polylactide nonwoven by using the layer-by-layer method in [17].

layer number	modifier	final structure
1	poly(acrylic acid) (PAA)	polylatide nonwoven-PAA- PDMAEMA-PAA- PDMAEMA-PAA
2	PDMAEMA	
3	poly(acrylic acid)	
4	PDMAEMA	
5	poly(acrylic acid)	

The authors of the work focus on solving one of the basic problems with the use of the LbL technique, namely, the introduction of the first charged layer. They consider the use of various acrylic acid grafting techniques as well as the entrapment of PAA macromolecules. After selecting the optimal method of starting the modification, the rest of the layers were applied using the LbL technique. The properties of the modified nonwoven fabric were confirmed using infrared reflection spectroscopy, potentiometric titration of acid groups, and measurement of the surface electric charge. It was found that by applying appropriate layers, it is possible to control the hydrophilicity, thermal properties and dyeability of the modified nonwoven fabric. Also in the work in [18], PDMAEMA is introduced on the surface of a polylactide nonwoven as one of the layers when modified with the LbL technique. The authors obtain complex structures on the surface of the nonwoven fabric by introducing the first layer by *in situ* polymerization of aniline. Samples with polyaniline show conductive properties and can also constitute the first layer for further modifications with the LbL technique. Different conductivity values are obtained depending on the number and type of successive layers. A paper [114] describes in detail the changes in the thermal characteristics of polypropylene nonwoven fabric modified with polyelectrolytes, including PDMAEMA. It was shown that the characteristic temperatures of thermal decomposition change depending on the type and number of layers applied. For example, in a system where there are two layers of PDMAEMA: PP nonwoven - PAA (grafted) - PDMAEMA - PAA - PDMAEMA, the temperature of 50% of thermal decomposition is about 30°C higher compared to the unmodified sample. The observed phenomenon takes place only in the presence of oxygen (the experiments were carried out in a synthetic air atmosphere).

### 4.6. Composite systems

Composite membranes made from poly(N,N-dimethylaminoethyl methacrylate) were developed using polysulfone and polyacrylonitrile substrates [115]. The deposited PDMAEMA layer was crosslinked by using p-xylylene dichloride. The obtained membrane including quaternary ammonium PDAEMA was analysed by typical analytical techniques. The polymer was formed into a thin dense layer on the substrates. The authors stressed that the quaternary and tertiary amino groups of PDMAEMA offered a high polarity and hydrophilicity.

## 5. Application of poly(N,N-dimethylaminoethyl methacrylate)

The preparation of commercially available materials (known under the trade name Eudragit) has already been reported [116]. Eudragit is a cationic copolymer based on PDMAEMA, butyl methacrylate, and methyl methacrylate. It is a powder used for the release of attached drugs in the stomach.

Gene transfer into intact cells is used nowadays to solve numerous problems in biology and medicine. In the last decades, gene delivery has received increasing interest as a possible therapy for treating a wide variety of diseases, including both genetic and non-genetic disorders [117–120]. Synthetic polyelectrolytes are known to be prospective agents for non-viral gene delivery. They have many benefits over other DNA transporting agents because of their ease of production, low risk of side effects, nonimmunogenicity and simplicity of DNA–polymer complex formation. Polycations and DNA form compact interpolyelectrolyte complexes in a solution by electrostatic bonds between negatively charged DNA phosphate groups and positively charged groups of polycations [72]. For some genetic diseases, gene therapy might be the ultimate cure. Dubruel & Schacht [121] explained that the mechanism is as follows: **‘***In the first step, a gene (i.e.* *a sequence of DNA nucleotides) is copied into messenger-RNA (m-RNA). This process takes place in the cell nucleus and is called transcription. Next, the m-RNA is transported from the cell nucleus to the ribosomes in the cytoplasm, where it acts as a matrix for the synthesis of a protein***’.** The practice explained above occurs for the synthesis of every protein required by a cell. However, when a mutation occurs in a gene, this can lead to a protein no longer being synthesized or to the incorrect synthesis of a particular protein. Dubruel & Schacht [121] stated: ‘*These phenomena can disrupt the correct functioning of cells. A possible solution for this problem can potentially be offered by gene therapy. In a classical gene therapy protocol, DNA, the carrier of the genetic information, is used as a drug for corrective treatment***’.** Polymers that have been applied most often as gene carriers are poly(α-amino acids) as poly(L-lysine) (PLL), linear and branched polyethyleneimine (PEI), poly(2-N,N-dimethylaminoethyl methacrylate) (PDMAEMA), DMAEMA-based (block) copolymers and terpolymers, other vinyl-based polymers, gelatin, and chitosan [121].

The main advantage of PDMAEMA-based gene delivery materials is that the transfection of PDMAEMA-based polyplexes is not serum dependent. This finding thus opens possibilities for the transfection of serum dependent cells. PDMAEMA was first applied as a non-viral gene delivery system in [27]—the polymer with a molecular weight of 360 000 was used. The relationship between size and charge of PDMAEMA/plasmid particles (Merck, Darmstadt, Germany) and their transfection efficiency was evaluated. Later, the same research group issued a more extended paper in which PDMAEMA with different molecular weights (4000–817 000) was compared. Van de Wetering *et al.* [24] discovered that the molecular weight of PDMAEMA significantly influenced the transfection efficiency and higher molecular weights (*M*_w_ > 300 000 g mol^−1^) showed higher transfection efficiencies. Also, it was reported in [22] that the molecular weight of PDMAEMA has a dramatic influence on the transfection efficiency, with gene expression increasing as a function of increasing molecular weight.

In [72], it can be found that the size and structure of PDMAEMA–DNA complexes are different depending on the solvent. The complex size does not exceed 50 nm. It was also shown that the transfection activity of PDMAEMA increases with average molecular weight; polymers with *M*_n_ > 300 000 have the maximal activity [24,72]. Also in [23], the transfection efficiency was studied as a function of the PDMAEMA molecular weight. Polymers with a molecular weight of 300 000 were better transfection agents than those with a low molecular weight. Dynamic light scattering tests showed that polymers with a high molecular weight were able to condense DNA effectively, resulting in particles with a size of 0.17–0.21 mm. On the other hand, when a plasmid was incubated with a low molecular weight polymer, large complexes were formed (size of approximately 1.0 mm). Evidently, smaller complexes have an advantage over larger complexes in terms of cell entry. Taking the results of the transfection efficiency and cytotoxicity together, we hypothesize that complexes enter cells by membrane destabilization, either at the cell surface or within vesicles.

PDMAEMA with a *M*_w_ of 1.6 **×** 106 g mol^−1^ and an Mn of 5.7 × 104 g mol^−1^ was used for gene transfer into human ovarian carcinoma cells in [122]. Another paper [77] described the application of linear, highly branched, and star-shaped PDMAEMA for gene transfer purposes. The authors found that the linear molecules showed the lowest transfection ability, while that of the branched molecules was slightly better; finally, the star-shaped PDMAEMA showed a significantly better transfection capability. Similar investigations of star-shaped polymers containing a cyclodextrin core and PDMAEMA arms were presented in [47]. This star structure exhibited a lower cytotoxicity compared to linear PDMAEMA. Different, practical aspects of using PDMAEMA as a DNA transfer can be found in many other papers [23–28,82,123,124]. A study [28] showed that the presence of sucrose, a low pH, and a low ionic strength favour the formation of relatively small polyplexes. In another paper [123], coating of PDMAEMA based polyplexes with a folate poly(ethylene glycol) conjugate led to a sharp decrease in the zeta potential and a small increase in the particle size. The other papers proved that the size of the polyplexes is an important factor for the transfection efficiency [23,24,27,124–126].

An interesting application of PDAEMA is its use as an element to increase the viscosity in lubrication systems [127]. The research shows that even a small proportion of the DMAEMA comonomer in the styrene-alkyl methacrylate copolymer has a significant impact on the viscosity and rheological properties of oils. The participation of DMAEMA in macromolecules significantly changes the molecular interactions between the polymeric additive and the oil, resulting in a substantially greater hydrodynamic volume of polymer molecules compared to conventional methacrylate additives. With an increase in the coil volume, the viscosity increases and the shear stability of the solutions decreases. The PDMAEMA polymers may find applications in micro- and nanotechnology. Gupta *et al.* [33] suggested applications of hydrogels as actuators, sensors, valves and other devices working based on the temperature and pH responsiveness of the analysed polymer.

### 5.1. Cross-linking of poly(N,N-dimethylaminoethyl methacrylate)

Cross-linking is the process of creating cross-links between macromolecules to obtain an insoluble network. The cross-linking mechanism may be different; the most common is the use of low-molecular cross-linkers with two functional groups (chemical cross-linking). High temperature (thermal) or radiation, most often UV (radiative), can also be used. The most popular cross-linking agents are di-halohydrocarbons, which can cross-link tertiary amino groups. Of course, the activity and molecular size of the agent have a large effect on the cross-linking reaction. For tertiary nitrogen alkylation in amine groups, the reactivity of the halogens follows the trend I > Br > Cl [128]. The reaction was completed in about 1 h at 80°C by using C_2_H_4_I_2_, but with Br derivatives, the reaction was very slow (at about 110°C). It is obvious that the structure of the halohydrocarbon also affects the cross-linking reaction; benzyl dihalides are more reactive than alkyl halides.

The excellent solubility of PDMAEMA, which has many advantages in terms of synthesis or processing, is very inconvenient from the application point of view. Therefore, PDMAEMA is often subjected to a cross-linking process prior to use, which causes the insolubility of the material. The finished polymer can be cross-linked, or the crosslinker can be added in one step during the polymerization. PDMAEMA can be crosslinked by using a heat energy treatment. For instance, in [129], the authors prepared a membrane using the PDMAEMA polymer as an active component in the form of a surface layer. Cuprammonium regenerated cellulose hollow fibre was a main substrate in this modification. The membrane was treated for 1 h at 40°C under vacuum conditions. Next, the temperature was increased to 120°C for 10 min in a heating dryer. The composite prepared in such a procedure was used for blood purification. A more complicated procedure was used in [130]. A composite membrane was obtained by depositing PDMAEMA from a water-ethanol solution on a non-woven polyester fabric (via a porous polyacrylonitrile layer). The evaporation procedure was performed for 2 min at 90°C, and the membrane was treated by heating (30 min at 125°C). The authors obtained a product with a PDMAEMA layer that was 3 µm thick.

In the work in [12], as a result of the cross-linking process, three-dimensional networks were obtained in which the degree of cross-linking is strictly dependent on the amount of cross-linker added to the monomer at the stage of polymerization. The authors also investigated the degradation of the obtained networks, stating that it depends both on the pH and the degree of cross-linking of the polymer; a higher cross-linking density resulted in faster degradation, so the process was easy to control. The use of the obtained networks, however, can be significantly limited because the obtained networks were found to have a cytotoxic effect on chondrosarcoma cells. In [15], the authors used the DMAEMA grafting method to introduce an antibacterial layer on the surface of silicone nanowires. Polymerization was performed using the ATRP technique. The polymer was then quaternized with benzoyl chloride. A system with a high antimicrobial efficiency against *Escherichia Coli* bacteria was obtained, and the time of active exposure was improved. A typical crosslinking agent such as p-xylylene dichloride was used in [131,132] to obtain a dense surface composite membrane for nanofiltration applications.

Hydrogels are a type of hydrophilic polymer that are cross-linked and absorb large amounts of water. Thanks to that, they can be a good vehicle for the storage or transport of other active species. Functional hydrogels have attracted much attention and can be treated as ‘smart’ polymer gels because they can change their properties in response to the external stimuli. These gels can alter the volume or shape by changing the pH, temperature, ionic strength, solvent composition or electric field. Swelling and shrinkage capacity are often easily reversible, and these materials have been proposed for various technological and biomedical applications, such as sensors, actuators, separation units, drug delivery systems, cartilage or even artificial muscles. PDMAEMA is a thermoresponsive polymer with a lower critical solution temperature of around 50°C. Due to the presence of amine groups, the polymer shows a pH sensitivity, giving it a wider area of uses [133]. In a previous paper [134], multi-responsible microgels based on PDMAEMA were developed and described.

Schallon *et al.* [77] synthesized cross-linked PDMAEMA for the purpose of gene transfer, while Gupta *et al.* [33] successfully created photocrosslinkable micro- and nanohydrogels of PDMAEMA. Those materials showed temperature- and pH-dependent swelling. The work in [73] presented the preparation of novel hydrogels based on PDMAEMA as a matrix and native or anionically modified potato starch as the entrapped polymer. Next, the authors investigated the influence of the DMAEMA concentration in the matrix and the structure and content of the polysaccharide. Gold nanoparticles coated with a PDAEMA layer were also used for cyanide sensing [36]. An effective colorimetric sensor using the detection of cyanide ions based on the dissolution mechanism was obtained.

### 5.2. Copolymers of poly(N,N-dimethylaminoethyl methacrylate) (selection)

The number of copolymers with DMAEMA units is very large. It is impossible to describe them all in one chapter. In order to collect them all, a separate work should be prepared devoted only to this issue. Therefore, only those examples of synthesis will be mentioned here which were subjectively considered by the author to be the most interesting. DMAEMA as a classical radical polymerization monomer can be co-polymerized with other monomers with similar reactivity factors. Different copolymers of DMAEMA can be used as flocculants (for beverages), suspensions in metallurgy, sea wages, elements of hair-care products, antistatic agents, and possibly as a soil conditioner or binder [50].

The work in [12] describes the synthesis of the DMAEMA-co-HEMA (HEMA: 2-hydroxyethyl methacrylate) copolymer. The authors of this work focused on the AIBN-catalyzed equimolar comonomer system. The polymerization was carried out under continuous spectroscopic (NMR) control. A random distribution of individual units in the synthesized copolymer was found. The copolymerization process of PDMAEMA with 4-cinnamoyphenyl methacrylate was shown in [134]:


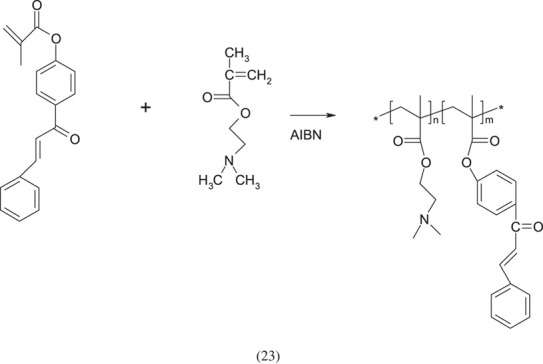


This copolymer was prepared by free radical copolymerization, and the reactivity ratios were determined with the extended Kelen-Tudos method. Fibre products made of poly-ε-caprolactone and DMAEMA diblock copolymers are described in [113]. This goal was achieved by electrospinning the mixed solutions. Both fibres and nonwovens were obtained and tested. The authors suggest using the materials for biomedical applications. Spasova *et al.* [135] showed the diblock copolymers polylacide-co-PDMAEMA. These copolymers were synthesized by ATRP of DMAEMA using the PLA-Br samples as macroinitiators:


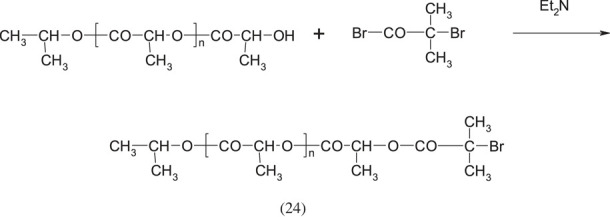


The synthesis was performed in THF at 60°C in the presence of a catalyst system and under a nitrogen atmosphere using a three-step procedure consisting of controlled ring opening polymerization of D-lactide or L-lactide initiated by aluminium triisopropoxide (Al(OiPr)_3_) to form R-isopropyloxy, ω-hydroxy polylactide (PLA-OH), followed by quantitative conversion of PLA-OH into R-isopropyloxy, ω-bromoisobutyratepolylactidemacroinitiator(PLA-Br), and ATRP polymerization of DMAEMA initiated by PLA-Br:


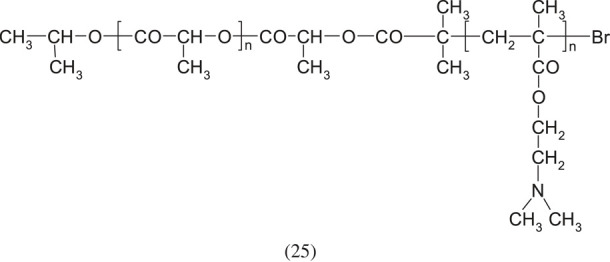


The author's interest for such diblock copolymers relies upon the presence of a biodegradable and biocompatible polylactide block that is able to form stereocomplexes and a water-soluble poly(aminomethacrylate) block known for its inherent biological activity. The presence of the PDMAEMA block enables the increase in the hydrophilicity of the PLA-based materials, as shown by water contact angle measurements. The authors suggested that these amphiphilic and adaptative copolymers are expected to pave the way for new materials that are potential candidates for biomedical applications such as wound healing and local cancer treatment.

DMAEMA copolymers with N-vinyl-pyrrolidone, methyl methacrylate and ethoxytriethylene glycol methacrylate were synthesized in [24]. Monomers were dissolved in toluene (final concentration 20%, w/v) and a stock solution of AIBN in the same solvent was added (M/I 100/1, mol mol^−1^). After 22 h at 60°C, the polymers were isolated by precipitation in 5–10-fold excess cold petroleum ether and dried *in vacuo*. The styrene-b-DMAEMA diblock copolymers were characterized in [136]. The authors obtained different sizes of individual blocks, suggesting the use of separations and drug delivery systems is possible.

Materials with bioactive properties are an important group of functional materials, and their range of applications is expected to expand in the future. Therefore, every new material is a valuable asset in engineering practice. Particularly significant are materials like PDMAEMA, which offer activity against a broad range of microorganisms, including gram-positive and gram-negative bacteria, viruses, and fungi. In the case of PDMAEMA, an additional advantage is achieved as these attractive properties already exist for the polymer with a tertiary nitrogen. Of course, quaternization further enhances the level of bioactivity. The challenge with implementing PDMAEMA on a larger scale is associated with the difficult polymerization process, which yields products with varying parameters and often gives non-reproducible results. Therefore, comparing the polyreactive processes will allow other researchers to design an optimal synthesis pathway.

Describing the various properties of PDAEMA will expand the range of applications for this polymer. From being a niche material known to a narrow group of researchers, it will become a much more common element in human future. It is a polymer that can be synthesized from both strictly linear macromolecules and those with different degrees of branching or cross-linking. With PDAEMA, it is possible to obtain films, fibers, particles, and use it as a standalone object or as a volumetric additive or modifying layer. It may be one of the materials of the future.

## 6. Conclusion

In this study, efforts were made to collect all relevant publications on PDMAEMA especially from recent years. It has been shown that this niche polymer, without a market producer, is quite popular. It is used relatively often due to its very interesting and specific properties. The most frequently used methods of carrying out the polymerization process, the forms in which the polymer can be used, the most important properties, and the areas of application are described. PDMAEMA has been described as a polyelectrolyte, a tertiary amine, and a quaternary ammonium salt. By using the data contained in the work, other researchers dealing with the introduction of this polymer to production or use will have the most important data collected in one document with source references. In this way, PDMAEMA should be more and more commonly used, and its very interesting properties will be more commonly used for the good of mankind.

## Data Availability

This article has no additional data.
